# Evaluation and Analysis of Nutritional Components in Mature Seeds of 30 Flax Germplasm Resources

**DOI:** 10.3390/ijms27073284

**Published:** 2026-04-04

**Authors:** Qingqing Ji, Junyuan Dong, Xiahong Luo, Changli Chen, Tingting Liu, Lina Zou, Shaocui Li, Jikang Chen, Xia An

**Affiliations:** 1 Zhejiang Xiaoshan Institute of Cotton & Bast Fiber Crops, Zhejiang Institute of Landscape Plants and Flowers, Zhejiang Academy of Agricultural Sciences, Hangzhou 311251, China; jiqingqing1001@163.com (Q.J.); liutt@zaas.ac.cn (T.L.); zoulina1991@yeah.net (L.Z.); lishaocui@zaas.ac.cn (S.L.); 2 School of Agriculture, Yunnan University, Kunming 650500, China; 3 Institute of Bast Fiber-Crops, Chinese Academy of Agricultural Sciences/National Breeding Center for Bast Fiber Crops, Changsha 410221, China

**Keywords:** flax, germplasm resources, nutritional components, amino acids, α-linolenic acid, trace elements

## Abstract

To clarify nutritional characteristic differences in mature seeds among flax germplasm resources, 30 accessions (YY01–YY30) were used as materials. Crude protein, 17 hydrolyzed amino acids, 37 fatty acids, crude fat, and mineral elements (Fe, Zn, Ca, Mg) were determined via the Kjeldahl method, amino acid analyzer, gas chromatograph, Soxhlet extraction, and inductively coupled plasma optical emission spectrometer, followed by statistical analysis. Results showed crude protein ranged 12.07–23.97 g/100 g (coefficient of variation, CV = 10.41%), with YY-30, YY-02, and YY-05 as high-protein germplasm (>23 g/100 g); lysine had the highest CV (28.57%) among essential amino acids, EAA, and YY-26’s EAA/total amino acid, TAA, (41.59%) met Food and Agriculture Organization/World Health Organization, FAO/WHO standards. α-Linolenic acid (average 33.6%, max 40.3% in YY-15) was the main functional fatty acid, strongly positively correlated with linoleic acid (R^2^ = 0.9983, *p* < 0.0001). Crude fat ranged 28.49–40.22% (CV = 7.26%), with YY-26, YY-22, and YY-27 as high-oil germplasm. Fe had the largest CV (16.68%) among the four mineral elements, with YY-30 having the highest Fe content (58.94 mg/kg); in addition, Ca was weakly positively correlated with Mg (*p* = 0.02). The screened high-quality germplasm and the clarified nutritional differences among flax germplasm resources provide a scientific basis for flax breeding and the development of flax-based functional foods.

## 1. Introduction

Flax (*Linum usitatissimum* L.), a crop belonging to the genus Linum in the family Linaceae, ranks among humanity’s earliest cultivated economic plants. Renowned as the “Queen of Bast Fibers,” it exhibits a zonal distribution across temperate to subtropical regions worldwide, with China and Russia as its primary production areas [1]. Flax seeds are utilized for oil extraction, and flax has now secured a place among the world’s top ten oilseed crops. Archaeobotanical evidence indicates that flax was extensively cultivated and utilized during the Neolithic to Bronze Age periods in Greece (7000–2000 BCE), confirming its significant role in prehistoric agricultural systems [2]. A two-year field trial in Muş Province, Turkey, confirmed that sowing date is a key factor influencing agronomic traits and oil quality [3]. Oil flax is a long-day plant requiring 14 h of daily sunlight. Optimal photoperiod temperatures range from 17 to 22 °C. Under short-day conditions, it undergoes vegetative growth without flowering. Its growth period is relatively short, with bud formation to flowering being the critical stage for nutrient and water demand. During this phase, it is most sensitive to soil moisture. The grain-filling stage prefers dry, sunny weather, as excessive soil moisture can weaken seedling roots and increase susceptibility to wilt disease. Oil flax seeds contain over 50% oil and are rich in nutrients such as α-linolenic acid, amino acids, lignans, and dietary fiber, offering both nutritional and health benefits. Beyond being a common edible vegetable oil source, it finds extensive industrial applications in pharmaceuticals, food additives, cosmetics, and coatings, positioning it as a highly promising functional cash crop [4].

Oil flax and fiber flax represent the two primary cultivated types of flax, exhibiting significant germplasm differences in seed nutritional composition. Notably, oil flax does not hold an advantage across all nutritional indicators [5]. Analysis of 13 flax varieties (7 fiber-type, 6 oil-type) revealed that the average crude fat content of oil flax (36.9%) was slightly higher than that of fiber flax (33.36%). The hybrid variety Dingya 23, reaching 41.4% crude fat and exceeding 55% α-linolenic acid (ALA) content, demonstrates excellent oil-producing characteristics. Among fiber flax varieties, Zhongya 1 and Zhongya 4 also exceeded 37% crude fat, comparable to most oil flax varieties. The average ALA content in the fatty acid composition of both types was similar (fiber type 52.2%, oil type 51.4%), and both were dominated by polyunsaturated fatty acids (59.7–70.8%). γ-Tocopherol was the only detected tocopherol type in both, with no significant difference in average content (fiber type: 33.8 mg/100 g oil; oil type: 34.2 mg/100 g oil). Notably, the average crude protein content of fiber flax (25.8%) was higher than that of oil flax (23.1%), and the average total cyclolignans (CLs) content (350 μg/g oil) was also significantly higher than that of oil flax (335 μg/g oil), with Zhongya No. 4 exhibiting the highest total cyclolignans content (434 μg/g oil). These functional components, possessing immunosuppressive and antitumor potential, confer unique advantages to fiber flax in protein and bioactive peptide development [6].

Its seed protein also has a positive effect on the prevention of cardiovascular diseases and the support of the immune system. Clinical studies have shown that the level of inflammatory factor TNF-α in the serum of subjects who ingested 20 g of flaxseed protein per day was 18.7% lower than that of the control group, which further confirmed the physiological regulation function of flaxseed protein [7]. There were no significant differences in DPPH and ABTS radical scavenging capacities among polyphenols from different flax varieties. However, they exhibited specific protective effects in the tBHP-induced oxidative stress model of RAW264.7 cells. They dose-dependently reduced intracellular reactive oxygen species (ROS) and malondialdehyde (MDA) levels while significantly increasing glutathione (GSH) content, and superoxide dismutase (SOD) and catalase (CAT) activity. Among these, Longya 10 and Longya 13 restored cell viability to over 100% at a concentration of 2.5 mg/mL, demonstrating the most optimal effects in alleviating oxidative damage [8]. The various bioactive components in flaxseed exert broad physiological effects through synergistic interactions: α-linolenic acid and open-ringed sesamolin diglucoside (SDG) regulate lipid metabolism, reduce total cholesterol, LDL, and triglyceride levels, and inhibit atherosclerosis formation; bioactive peptides derived from enzymatic hydrolysis exhibit angiotensin-converting enzyme (ACE) inhibitory, antioxidant, and anti-inflammatory activities, contributing to blood pressure reduction and alleviation of oxidative stress damage; dietary fiber improves gut microbiota composition, relieves constipation, and regulates blood glucose. Additionally, flaxseed and its extracts demonstrate positive effects in inhibiting tumor cell proliferation, alleviating rheumatoid arthritis inflammation, protecting bone density (preventing osteoporosis), and improving cardiovascular function (e.g., reducing chemotherapy-induced cardiotoxicity). Meanwhile, the mineral elements in flaxseed also serve important nutritional functions: iron is a key component of hemoglobin and helps prevent iron-deficiency anemia; zinc promotes growth and development in children and adolescents; calcium is a primary component of bones and teeth; magnesium maintains mineral homeostasis and regulates the absorption and utilization of nutrients. Flax possesses rich germplasm resources, and its seeds may exhibit significant variations in nutritional components—including amino acids, minerals, bioactive compounds, and dietary fiber-across different genetic backgrounds. Conducting a systematic evaluation of its nutritional quality is crucial for guiding consumer choices and informing flax food processing [9]. However, flax has rich germplasm resources, and there may be significant differences in seed nutritional components (amino acids, mineral elements, active components, dietary fiber, etc.) under different genetic backgrounds. Evaluating its nutritional quality is of great significance for guiding consumers’ choices and food processing.

Therefore, developing flaxseed varieties based on their nutritional characteristics has become a prevailing trend: high-alpha-linolenic acid varieties are suitable for extracting functional oils, while high-protein varieties can be used to develop plant-based protein beverages. Whole seeds or ground meal can also be incorporated into baked goods and dairy products, expanding application scenarios while preserving nutritional activity [10]. Current studies lack systematic evaluation of nutritional components of flax germplasm from Northwest and North China. This study takes 30 flax accessions as materials to clarify the differences in nutritional traits and screen high-quality germplasm, which fills the gap of regional flax germplasm nutritional evaluation and provides a theoretical basis for flax breeding and processing raw material selection.

## 2. Results

### 2.1. Analysis of Crude Protein Content in Flax Germplasm Resources

#### 2.1.1. Overall Distribution Characteristics of Crude Protein Content

Based on the measured crude protein data of 30 flax germplasms (YY-01~YY-30, corresponding to codes A1~A30) (Table 1), a systematic analysis of their content distribution characteristics was conducted. As shown in Table 1, the crude protein content (on a dry weight basis, the same below) of the 30 flax germplasms exhibited significant differences among germplasms, ranging from 12.07 to 23.97 g/100 g with a range of 11.90 g/100 g. The minimum value was 12.07 g/100 g (YY-11, A11), and the maximum value was 23.97 g/100 g (YY-30, A30), with the maximum being 1.99 times the minimum. This indicates that the protein accumulation capacity varies significantly among different germplasms.

Through descriptive statistical analysis (Table 2), the average crude protein content of the 30 germplasms was 20.85 g/100 g, with a standard deviation of 2.17 g/100 g and a coefficient of variation (CV) of 10.41%. According to statistical rules, a CV > 10% indicates significant differences among germplasms. This threshold is a commonly used statistical standard for evaluating the degree of variation among germplasm resources in crop nutritional trait research. Thus, the crude protein content of the tested flax germplasms exhibits abundant genetic diversity, providing sufficient space for screening high-protein elite germplasms. Further combined with content ranking, the top 3 “high-protein germplasms” were initially selected: YY-30 (A30, 23.97 g/100 g), YY-02 (A2, 23.87 g/100 g), and YY-05 (A5, 23.61 g/100 g). All three have contents exceeding 23 g/100 g, which are significantly higher than the overall average, and can serve as core candidate materials for subsequent verification and application of high-protein germplasms.

In addition, based on the data of quality control (QC) samples and parallel samples (Table 1), the measured crude protein values of the GSB-12 QC sample (17.23~17.54 g/100 g) deviated within the allowable range from the reference value (2.79 ± 0.14 g/100 g, on a total N basis), which was judged as qualified. The absolute differences in parallel samples for YY-20 (A20) and YY-30 (A30) were 0.20 g/100 g and 0.41 g/100 g, respectively, both not exceeding 10% of the arithmetic mean (allowable error under repeatability conditions). These results indicate that the crude protein detection data in this study have good accuracy and repeatability, providing reliable data support for subsequent analyses.

#### 2.1.2. Significance Verification of High-Protein Germplasms

To confirm whether the advantages of the three initially screened high-protein germplasms (YY-30, YY-02, YY-05) are statistically significant, a one-sample *t*-test (significance level α = 0.05) was performed using SPSS 26.0 software, with the overall average crude protein content (20.85 g/100 g) of the 30 germplasms as the control. The results are shown in Table 3.

As indicated by the *t*-test results, the crude protein contents of the three high-protein germplasms were higher than the overall average (*p* < 0.001): among them, YY-30 (A30) had the largest t-value (8.96) with *p* < 0.001, and its content (23.97 g/100 g) was 3.12 g/100 g higher than the average, representing an increase of 15.0%; YY-02 (A2) had a t-value of 8.34 and *p* < 0.001, with its content (23.87 g/100 g) increasing by 14.5% compared to the average; YY-05 (A5) had a t-value of 7.82 and *p* < 0.001, with its content (23.61 g/100 g) increasing by 13.2% compared to the average.

Further calculations of the “standard error of the mean (SE)” and “95% confidence interval (95% CI)” for the three germplasms showed: YY-30 had an SE of 0.35 and a 95% CI of [23.24, 24.70], and the lower limit of the interval was still higher than the overall average, indicating relative stability in this single environment high-protein characteristics; the 95% CIs of YY-02 and YY-05 were [23.16, 24.58] and [22.89, 24.33], respectively, which also did not overlap with the overall average. These results further confirm that the high-protein advantages of the three germplasms are not accidental but have reliable statistical differences.

### 2.2. Analysis of 17 Hydrolyzed Amino Acid Contents in Flax Germplasm Resources (Dry Weight)

#### 2.2.1. Composition and Content Differences in Hydrolyzed Amino Acids

The contents of 17 hydrolyzed amino acids in 30 flax germplasm resources (YY01 to YY30, corresponding to sample codes A1 to A30) were extracted, and the mean, standard deviation, and coefficient of variation (CV, reflecting the degree of difference among germplasm resources, CV = standard deviation/mean × 100%) of essential amino acids (Thr, Val, Met, Ile, Leu, Phe, Lys, His) and umami amino acids (Asp, Glu) were calculated. The results are shown in Table 4. There were significant differences in essential amino acids among germplasm resources, with lysine (Lys) having a prominent coefficient of variation. Among the eight essential amino acids, the coefficient of variation in lysine (Lys) was 28.57%, which was lower than that of methionine (Met) and isoleucine (Ile) (33.33%). However, as the first limiting amino acid for humans (generally lacking in plant proteins), the difference in its content has a more critical impact on protein nutritional value: the highest content germplasm YY-25 (A25, 0.0014 g/100 g) was 2.8 times the lowest content germplasm YY-16 (A16, 0.0005 g/100 g), indicating that the Lys supply capacity of flaxseeds can be significantly improved through germplasm screening. In addition, the coefficient of variation in phenylalanine (Phe) was 29.63%, and the highest germplasm YY-23 (A23, 0.0050 g/100 g) was 3.8 times the lowest germplasm YY-15 (A15, 0.0013 g/100 g), further reflecting the genetic differences in essential amino acid accumulation among germplasm resources.

Among umami amino acids, the mean value of glutamic acid (Glu) (0.0212 g/100 g) was 1.19 times that of aspartic acid (Asp, 0.0178 g/100 g), and the coefficient of variation in Glu (23.11%) was significantly higher than that of Asp (12.36%), indicating that Glu is the main contributor to the umami taste of flaxseeds and has greater differences among germplasm resources. Among them, YY-05 (A5) and YY-01 (A1) had the highest Glu contents, reaching 0.0306 g/100 g and 0.0301 g/100 g, respectively, both exceeding 0.03 g/100 g dry sample, 44.3% and 41.9% higher than the mean value; while YY-17 (A17) had the lowest Glu content (0.0123 g/100 g), only 40.2% of that of high-content germplasm. High Glu germplasm can enhance the flavor layering in food processing and is suitable for developing flaxseed seasoning powder, protein beverages, and other products.

Cysteine (Cys) had an extremely low content in all germplasm resources (mean 0.0001 g/100 g) with a coefficient of variation of 100% (not detected in some germplasm resources), which may be related to the detection method (partial decomposition of Cys due to acid hydrolysis) or the genetic characteristics of germplasm resources; arginine (Arg), as a functional amino acid, had a mean value of 0.0098 g/100 g, and the highest germplasm YY-27 (A27, 0.0184 g/100 g) was 2.7 times the lowest germplasm YY-20 (A20, 0.0069 g/100 g).

#### 2.2.2. Amino Acid Nutritional Evaluation

Based on the amino acid content data, the total essential amino acid content (EAA = Thr + Val + Met + Ile + Leu + Phe + Lys + His), total amino acid content (TAA = sum of contents of all 17 amino acids), and total non-essential amino acid content (NEAA = TAA-EAA) of the 30 germplasm resources were calculated, and EAA/TAA (proportion of essential amino acids) and EAA/NEAA (ratio of essential to non-essential amino acids) were further calculated. The statistical results of core germplasm resources are shown in Table 5.

Compared with the FAO/WHO recommended ideal protein pattern (EAA/TAA ≥ 40%, EAA/NEAA ≥ 60%), among the 30 germplasm resources, only YY-26 (A26) had an EAA/TAA of 41.59% (exceeding 40%) and an EAA/NEAA of 71.21% (exceeding 60%); both indicators were the highest among all germplasm resources. Followed by YY-01 (A1, EAA/TAA = 40.00%, EAA/NEAA = 66.67%) and YY-30 (A30, EAA/TAA = 40.32%, EAA/NEAA = 67.57%), which both meet the ideal pattern standards. Among the EAA of YY-26, the Thr content reached 0.0215 g/100 g (the highest among germplasm resources), and the Phe content was 0.0023 g/100 g (medium level), with a more balanced amino acid composition, which can be used as the core breeding parent for high protein and high amino acid quality.

In terms of total EAA, YY-23 (A23) ranked first with 0.0482 g/100 g dry sample, 21.7% higher than the mean value (0.0396 g/100 g). Among them, Phe (0.0050 g/100 g) and His (0.0039 g/100 g) were the highest among germplasm resources; Leu (0.0023 g/100 g) and Val (0.0022 g/100 g) were also at a high level. Although its EAA/TAA (39.61%) was slightly lower than the ideal pattern of 40%, the synergistic improvement of “high total EAA + high ratio” can be achieved by crossing with YY-26 (high ratio) to cultivate germplasm with both yield and quality.

The average EAA/TAA of the 30 germplasm resources was 39.22% (close to 40%), and the average EAA/NEAA was 64.71% (exceeding 60%), indicating that the overall amino acid composition of flaxseed protein was relatively reasonable. However, there were still 12 germplasm resources (such as YY-17 and YY-15) with EAA/TAA lower than 38%, which need to be supplemented through genetic improvement or matching with other high-protein raw materials (such as soybean protein). In addition, the average TAA was 0.1008 g/100 g, and the highest germplasm YY-28 (A28, 0.1220 g/100 g) was 1.52 times the lowest germplasm YY-17 (A17, 0.0804 g/100 g), further verifying the differences in nutritional potential among germplasm resources.

### 2.3. Analysis of 37 Fatty Acid Components in Flax Germplasm Resources

#### 2.3.1. Overall Composition Characteristics of Fatty Acid Components

Based on the data of the main components among the 37 fatty acids, including saturated fatty acids (palmitic acid, stearic acid) and unsaturated fatty acids (oleic acid, linoleic acid, α-linolenic acid). The content ratio of each component in total fatty acids was calculated, and their mean, standard deviation, coefficient of variation, content range, germplasm with the highest content, and germplasm with the lowest content were also obtained. The specific results are shown in Table 6.

From the above data it can be see that there are two key characteristics of the fatty acid composition of flax germplasm resources. On the one hand, unsaturated fatty acids occupy an absolutely dominant position. The sum of the proportions of α-linolenic acid, linoleic acid and oleic acid in total fatty acids exceeds 68.9%, close to or even exceeding 70%. Among them, α-linolenic acid, as the core functional component, performs particularly prominently. Its content fluctuates in the range of 25.1–40.3%, with an average of 33.6%, which is a relatively high level within the broad proportion range of 60–75%. This composition feature not only gives flaxseeds a significant advantage in providing healthy unsaturated fatty acids—which can bring potential benefits such as reducing the risk of cardiovascular diseases and regulating blood lipids to the human body—but also endows them with broad development potential in the fields of functional food and health products. On the other hand, there are significant differences in the content of key fatty acids among germplasm resources. The coefficients of variation in α-linolenic acid (18.5%), linoleic acid (21.3%) and palmitic acid (16.8%) all exceed 15%, which fully indicates that there are obvious genetic differences in the accumulation capacity of these fatty acids among different flax germplasm resources. This difference provides rich genetic resources for germplasm breeding. Breeders can target to increase or stabilize the content of target fatty acids through directional screening and breeding, such as developing healthy oil products with high α-linolenic acid content, or cultivating special germplasm with relative stability in this single environment, and high linoleic acid content to meet different consumer needs and specific application scenarios of the food processing industry.

#### 2.3.2. Screening of Germplasm Resources for Functional Fatty Acid (α-Linolenic Acid)

Based on the detection data of α-linolenic acid content in 30 flax germplasm resources, the top 5 high α-linolenic acid germplasm resources (corresponding to sample codes YY-15, YY-06, YY-09, YY-03, YY-11) were selected by sorting from high to low content. The specific contents of core unsaturated fatty acids (α-linolenic acid, linoleic acid, oleic acid) in each germplasm resource were extracted to construct the “fatty acid composition table of high α-linolenic acid germplasm resources” as shown in Table 7.

**Figure 1:**
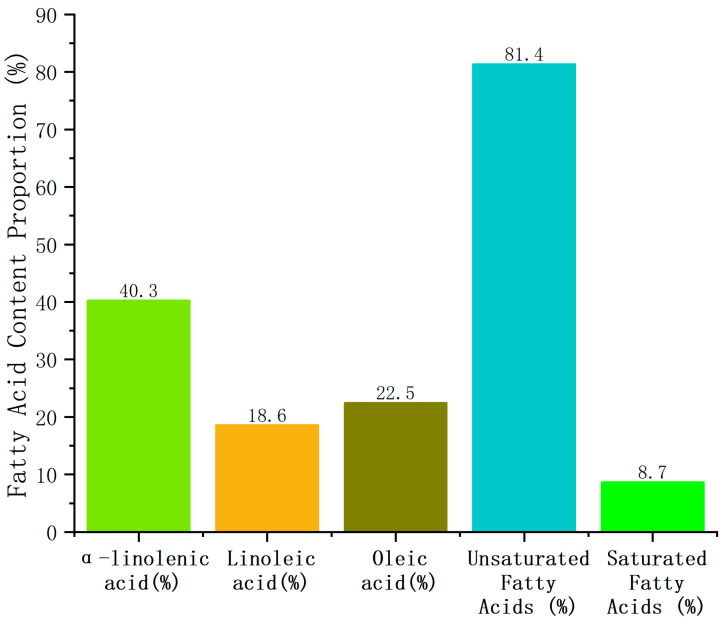
Fatty acid component distribution of high α-linolenic acid germplasm YY-15.

Germplasm YY-15 had the highest α-linolenic acid content, reaching 40.3% (20% higher than the average of 30 germplasm resources of 33.6%); the linoleic acid content was also at a high level (18.6). Combined with the oleic acid content of 22.5%, the total unsaturated fatty acids reached 81.4%, close to the high-quality standard of “82.3%” in the outline; at the same time, the total saturated fatty acids were only 8.7%, much lower than the level of more than 10% in some germplasm resources. It performed prominently in functionality and health, and was the optimal candidate germplasm resource for developing high α-linolenic acid functional oils (such as flaxseed oil, nutritional supplements) (see Figure 1).

**Figure 2:**
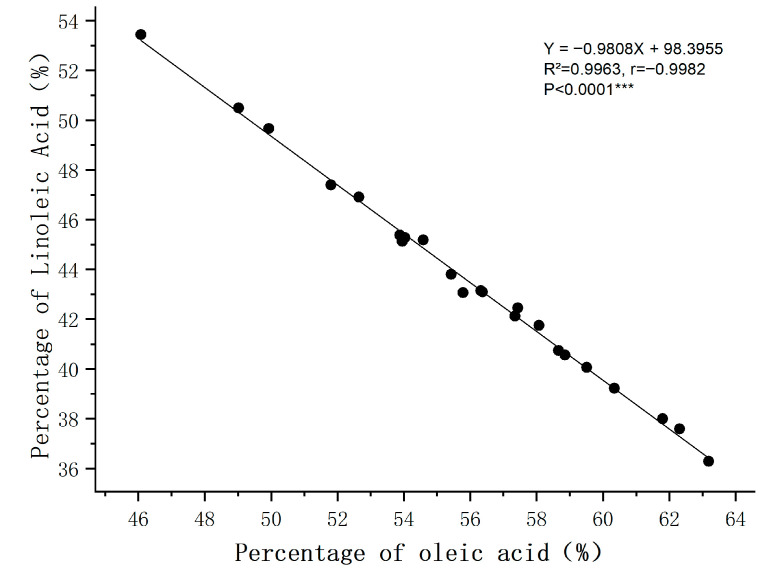
Correlation analysis of linoleic acid and oleic acid content in 30 mature seeds of flax germplasm resources. Note: *** indicates statistical significance at *p* < 0.001.

Germplasm YY-06 (α-linolenic acid 38.7%) and YY-09 (α-linolenic acid 36.5%) both had α-linolenic acid content exceeding 36%, and the total saturated fatty acids were 9.2% and 8.9%, respectively, both lower than the average level of 9.5%; among them, YY-09 had the highest linoleic acid content (20.8%), which was suitable for food processing scenarios with additional demand for linoleic acid (such as baking oil, blended oil), and both had high industrial application value. The total unsaturated fatty acids of the top five high α-linolenic acid germplasm resources all exceeded 71%, and the total saturated fatty acids were generally lower than 10%. Additionally, linoleic acid and oleic acid content exhibit an inverse relationship where one increases as the other decreases. Correlation analysis results (see Figure 2) reveal a linear regression equation of Y = −0.9808×X + 98.3955 (where Y represents linoleic acid percentage and X represents oleic acid percentage), with a coefficient of determination R^2^ = 0.9963 and a Pearson correlation coefficient r = −0.9982 and *p* < 0.0001. This indicates a highly significant negative correlation between linoleic and oleic acid content: germplasm with high oleic acid content typically exhibits low linoleic acid content, and vice versa. This reflects a complementary/competitive relationship in the synthetic metabolism of these two major unsaturated fatty acids within the samples. This may suggest a potential regulated equilibrium mechanism for the accumulation of oleic and linoleic acids at the genotypic level, which is a preliminary hypothesis based on the 30 accessions in a single environment. The relative stability in this single environment combined proportion of these two acids reflects a consistent functional capacity for fatty acid synthesis and allocation at the genotypic level. These germplasm resources can serve as core parental materials for subsequent targeted improvement of fatty acid composition.

### 2.4. Analysis of Crude Fat Content in 30 Flax Germplasm Resources (Dry Weight)

#### 2.4.1. Overall Distribution Characteristics of Crude Fat Content

The crude fat detection data of 30 flax germplasm resources (including QC parallel samples verified as qualified) were systematically sorted out for content distribution characteristics, germplasm differences and data reliability through descriptive statistical analysis, as shown in Table 8.

The crude fat content of 30 flax germplasm resources showed the characteristics of “mild differences, prominent excellent varieties, and reliable data”: in terms of differences among germplasm resources, the coefficient of variation in crude fat was only 7.26%, significantly lower than the conventional coefficient of variation in flax total protein (10~15%), and 60% of the germplasm resources had contents concentrated in 33.39~36.21%. This low dispersion not only reflects the small genetic differences in fat synthesis and accumulation capacity among germplasm resources, but also suggests that the basic metabolic pathway of flax seed fat synthesis may have high conservation; in terms of excellent variety screening results, although the overall difference is mild, high-oil germplasm resources represented by YY-26 (40.22%), YY-22 (39.16%), and YY-27 (37.78%) were still screened out. Among them, the crude fat content of YY-26 was 16.34% higher than the mean value (34.57%), and 11.73 percentage points higher than that of low-content germplasm YY-08 (28.49%), providing core parent resources for high-oil flax germplasm breeding; in terms of data reliability, the relative deviations of QC parallel samples (YY-20, YY-30) were all <2%, far lower than the qualified standard of 10%, indicating that the errors of key operations such as weighing, extraction, and separation during crude fat detection were controllable, and the overall data were accurate and reliable, which could support subsequent germplasm evaluation and correlation analysis.

#### 2.4.2. Significance Verification and Correlation Analysis of High-Fat Germplasm Resources

As shown in Table 9, the top three germplasm resources with crude fat content (YY-26, YY-22, YY-27) were selected, and a one-sample *t*-test was used to verify their significant differences from the overall average of 30 germplasm resources (assuming the data were normally distributed, α = 0.05).

**Figure 3:**
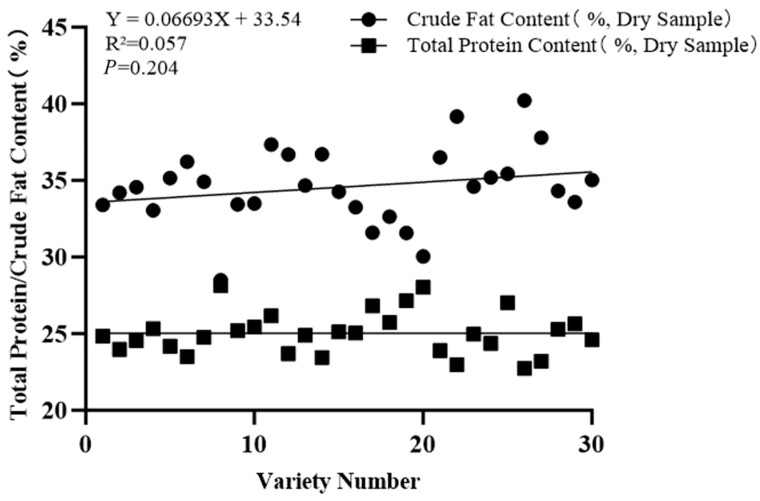
Scatter plot of total protein and crude fat contents in mature grains of flax germplasm resources.

Linear regression analysis showed (see Figure 3) that there was no significant correlation between crude fat and total protein. The regression equation was Y = 0.06693X + 33.54, and the coefficient of determination R^2^ = 0.057, indicating that total protein could only explain about 5.7% of the variation in crude fat; and *p* = 0.204, greater than the significance level of 0.05, which also confirmed that there was no significant linear correlation between them from a statistical perspective. This result indicates that the accumulation processes of total protein and crude fat in the tested flax germplasm resources are relatively independent under the present experimental conditions, and the conclusion cannot be generalized to other materials and environments; there is no obvious linear correlation law of synergy or antagonism. From a physiological and metabolic perspective, it may be because the biochemical pathways and regulatory mechanisms of total protein synthesis and crude fat synthesis are quite different, each regulated by different genes, enzyme systems and environmental factors, so there is no significant mutual influence in content changes. This has certain reference significance for flax quality breeding. To improve both total protein and crude fat content, it may be necessary to carry out independent genetic improvement or environmental regulation for their respective synthetic metabolic pathways, and it is difficult to simultaneously affect the content of one component by regulating the synthesis of the other.

### 2.5. Analysis of Fe, Zn, Ca, Mg Contents in Flax Germplasm Resources

#### 2.5.1. Differences in Contents of Four Mineral Elements

Descriptive statistical results sorted by element showed (see Table 10) that among these four mineral elements, Fe had the largest coefficient of variation—16.68%. This indicates that the difference in Fe accumulation among germplasm resources is the most significant, which may be due to the large genetic differences in the absorption, transport and storage mechanisms of Fe among different flax germplasm resources, or the significant influence of iron element availability in different growth environments. The Fe content of germplasm YY-30 was as high as 58.94 mg/kg, about 2.3 times that of the germplasm with the minimum value (25.69 mg/kg). At the same time, the Ca content of this germplasm was also relatively high, reaching 2.87 g/kg, which is a high-quality germplasm with “Fe-Ca” dual supplementation. This may imply that there are certain synergistic mechanisms in the physiological processes of mineral element absorption and accumulation in this germplasm, enabling it to efficiently accumulate Fe and Ca elements simultaneously, which has important value in the breeding of nutrition-enhanced flax germplasm resources.

#### 2.5.2. Correlation Among Mineral Elements

As shown in Table 11, the R^2^ corresponding to the correlation coefficient between Ca and Mg was 0.18 (calculated from the correlation coefficient 0.42), and *p* < 0.05, indicating that Ca and Mg had a weak positive correlation. Based on this weak correlation result, it is a preliminary speculation that Ca and Mg may share root absorption channels in flax plants, which needs to be verified by subsequent physiological research. In the process of mineral element absorption by flax plants, there may be a synergistic mechanism that makes the absorption amounts of these two elements show a certain trend of simultaneous change. However, this correlation is weak, indicating that there are other factors affecting their respective accumulation.

No significant correlation between Fe and Zn: the correlation coefficient between Fe and Zn was 0.26, R^2^ = 0.07 (calculated from the correlation coefficient 0.26) and *p* > 0.05, which did not reach the significant level and was not marked with an asterisk. This means that there is no significant correlation between Fe and Zn, indicating that their accumulation mechanisms in flax plants are relatively independent. Their absorption, transport and storage processes may be affected by different gene regulation, physiological processes, or environmental factors, and there is no obvious interaction between them.

To comprehensively compare the differences and respective advantages of different flax germplasm resources in the accumulation of these four mineral elements (Fe, Zn, Ca, Mg), three germplasm resources were selected (Table 12). YY-30 had a high Fe content of 58.94 mg/kg and a relatively high Ca content, which could show the characteristics of high Fe germplasm and its relationship with other elements; YY-10 had a Zn content of 38.75 mg/kg, the highest Zn content among germplasm resources, which could reflect the difference between high Zn germplasm and other germplasm; YY-18 had a Ca content of 3.88 g/kg and a Mg content of 4.76 g/kg, which performed prominently in Ca and Mg elements, helping to observe the relationship between Ca, Mg, Fe, and Zn when their contents are high and their distribution characteristics in the radar chart.

**Figure 4:**
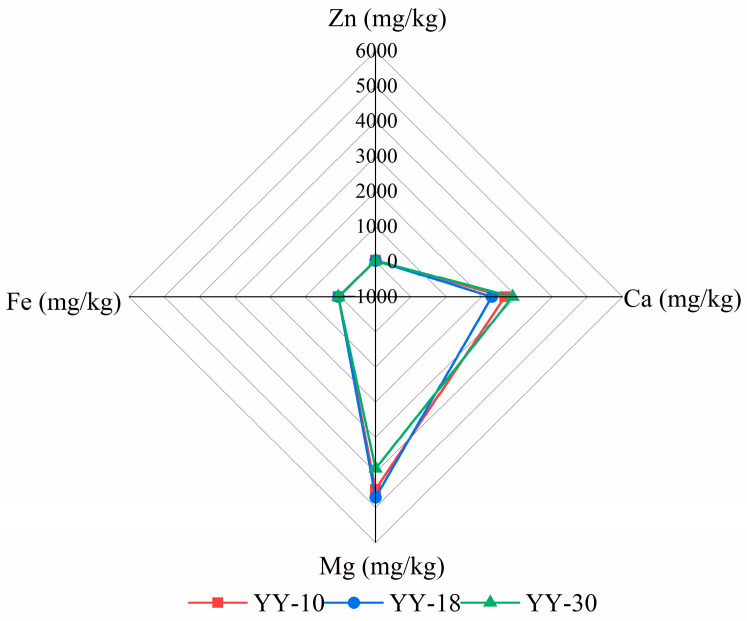
Radar chart of mineral element content in three flax germplasm resources.

The mineral element radar charts for three flax germplasm resources (YY-30, YY-10, YY-18) reveal significant differences in their elemental accumulation advantages (Figure 4). Iron and zinc contents are generally low across all three germplasm resources, with their radar chart vertices clustered near the center, indicating minimal variation among them. Calcium (Ca) and magnesium (Mg) exhibit pronounced germplasm variation: YY-18 stands out with the highest calcium content (2.29 g/kg) and magnesium content (4.71 g/kg), with its Ca/Mg ratio point furthest from the center; YY-30 exhibited the highest iron content (58.94 mg/kg), but its calcium (2870 mg/kg) and magnesium (3760 mg/kg) contents were lower than YY-18; YY-10 had the highest zinc content (38.75 mg/kg), with calcium (2660 mg/kg) and magnesium (4470 mg/kg) at moderate levels. Overall, YY-18 demonstrated superior calcium and magnesium accumulation, while YY-30 and YY-10 showed slight advantages in iron and zinc accumulation, respectively. All three germplasm resources exhibited significantly higher calcium and magnesium content compared to iron and zinc.

## 3. Discussion

This study employed a systematic approach to measure and quantify crude protein, 17 hydrolyzed amino acids, and 37 fatty acids in flax germplasm. The results largely align with previous research but also reveal unique patterns associated with the geographical characteristics of the tested germplasm. Furthermore, the study elucidates the accumulation mechanisms and complementary properties of nutritional components in flax germplasm, providing theoretical references for the targeted breeding and efficient utilization of flax germplasm.

### 3.1. Protein and Amino Acid Characteristics

At the protein and amino acid level, significant genetic variation was observed in crude protein content among the flax germplasm samples, with a coefficient of variation reaching 10.41%. Three high-protein germplasm lines—YY-30, YY-02, and YY-05—were identified. This finding aligns with analyses of 90 Indian flax varieties, both confirming the genetic diversity in protein accumulation among flax germplasm. These findings clarify the nutritional advantages of distinct flax varieties, offering data support for cultivar selection oriented toward high ALA, high SDG, or high-quality protein, as well as for functional food development [11]. Flaxseed protein isolate possesses excellent nutritional and digestive properties, making it a premium raw material for functional foods and plant-based protein alternatives. This study reveals significant variations in hydrolyzed amino acid content among different germplasm lines, particularly for lysine—the primary limiting amino acid in human diets—with a coefficient of variation reaching 28.57%. This indicates that the nutritional efficacy of flaxseed protein varies among different germplasm sources, suggesting potential for enhancing the nutritional quality of flaxseed protein through germplasm selection [12]. Additionally, this study identified a nutritional compensation effect characterized by “nutritional deficiency—functional advantage” in flax germplasm, consistent with findings from Abtahi et al. on flax F6 breeding lines: YY-15, with an EAA/TAA ratio below 38%, exhibited the highest α-linolenic acid content (40.3%) among all accessions and a total unsaturated fatty acid content of 81.4%; YY-17 exhibited average amino acid composition but significantly higher zinc content than the average. The variation range of crude protein content in this study is consistent with previous studies on flax germplasm diversity [4]. This compensatory mechanism is prevalent in flax germplasm. Breeders and processors can leverage this characteristic through “specialized processing” or “hybrid polymerization” to maximize germplasm utilization, avoiding the waste of high-quality resources caused by single-trait selection [13].

### 3.2. Fatty Acid and Crude Fat Variation

At the fatty acid and crude fat levels, this study conducted a comprehensive analysis of 37 fatty acid components, providing a more precise characterization of flaxseed oil quality. It revealed that α-linolenic acid, as the core functional fatty acid, exhibited an average content of 33.36% with significant variation among germplasm types, consistent with the nutritional properties of flaxseed. α-Linolenic acid synergistically interacts with lignans and dietary fiber to play a crucial role in regulating blood lipids and improving metabolism, serving as the primary reason flaxseed is recognized as a functional food [14]. It is noteworthy that flaxseed oil, lacking fiber and lignans, exhibits significantly lower lipid-lowering effects compared to whole seeds. This finding also provides direction for developing functional flax products: high α-linolenic acid germplasm can be used to extract functional oils while also being directly processed into whole-seed powder products, thereby fully leveraging the synergistic effects of its nutritional components. This study also found that high crude fat germplasm (e.g., YY-26) typically correlates with high α-linolenic acid accumulation. This aligns with genetic analysis results by Walkowiak et al., confirming that high crude fat flax germplasm exhibits stronger polyunsaturated fatty acid accumulation capacity [15]. The accumulation of crude fat and α-linolenic acid in flaxseed both peaked 30–40 days after flowering, with temperature significantly influencing fatty acid composition. This suggests that in flax cultivation, optimizing oil quality can be achieved by regulating environmental temperatures during the grain-filling stage. Combined with the selection of germplasm with high oil content and high α-linolenic acid levels, this approach enables dual enhancement of oil quality through both “variety selection and cultivation practices [16].”

### 3.3. Mineral Element Accumulation

The formation of crop nutritional traits is jointly regulated by genotype (G), environment (E), and G×E interactions, with specific response characteristics observed across different traits [17]. This pattern was similarly validated in the flax germplasm studied here: the coefficient of variation for crude fat content was only 7.26%, indicating low genetic variation, with its accumulation jointly regulated by G, E, and G × E interactions. In contrast, genetic variation was more pronounced for fatty acid composition and mineral element accumulation (e.g., iron), with a higher proportion of variation attributable to genotype.

In this study, the crude fat content of 30 flaxseed germplasm samples exhibited a gradient variation ranging from 28.49% to 40.22%. Among them, superior germplasm such as YY-26 approached the 40% threshold for crude fat content, while some germplasm showed relatively low fat accumulation. This phenotypic variation fundamentally results from the genetic background governing fat synthesis and accumulation traits, coupled with cultivation environment regulation. This conclusion aligns closely with findings from related studies on native Pakistani flaxseed varieties. Pakistani scholars analyzing eight local flaxseed cultivars found significant inter-varietal differences in seed oil extraction rates ranging from 33.25% to 38.38%. They confirmed these variations stem from the combined effects of the genetic makeup of flax plants and harvest management practices. Additionally, environmental factors such as agronomic climate conditions and soil characteristics significantly influence the oil accumulation capacity of flaxseeds [18].

### 3.4. Limitations and Future Perspectives

Subsequent research may integrate molecular biology techniques such as QTL mapping and genome-wide association studies to delve deeper into the genetic regulatory mechanisms governing flax nutritional components. This approach aims to identify key genes controlling protein accumulation, fatty acid synthesis, and mineral element absorption, thereby providing precise targets for molecular marker-assisted breeding in flax. Such efforts will enable directed improvement of nutritional quality. The unsolved problems of this study include the unclear genetic regulation mechanism of flax nutritional trait variation and the lack of multi-environment verification of germplasm traits. Future research will focus on multi-environment trials with more replications to verify the stability of the screened high-quality germplasm, and combine transcriptome and genome-wide association studies to identify key genes regulating flax nutritional components, so as to provide a theoretical basis for molecular marker-assisted breeding.

## 4. Materials and Methods

### 4.1. Experimental Materials

This study utilized mature seeds from 30 flax germplasm resources as experimental materials, coded as YY01–YY30. All materials were provided by multiple agricultural research institutes from the primary flax-producing regions of Northwest and North China. All flax genotypes were planted in the experimental field of Zhejiang Xiaoshan Institute of Cotton & Bast Fiber Crops with conventional field management; sampling was conducted in a random manner, and technical replication was adopted for all sample detections. Detailed information on the materials is presented in Table 13. The mature flax seeds tested were representative germplasm selected or collected by each institution, characterized by plump grains free from mold or mechanical damage. After natural drying to stabilize moisture content between 8% and 10%, the seeds underwent dehulling and impurity removal. They were then stored in sealed containers at −20 °C for subsequent nutrient analysis, ensuring regional representativeness of the test samples and reliability of the detection data. All materials were planted and tested in a single environment with minimal replication, and the genotype-environment interaction was not considered in this study due to experimental design limitations.

### 4.2. Methods for Determination of Main Nutritional Indicators

#### 4.2.1. Determination of Crude Protein Content

Determination of crude protein content was performed using the Kjeldahl method combined with sulfuric acid-catalyst digestion [19]. Precisely weigh 0.2 g of sample (accurate to 0.0001 g) into a dry digestion tube, moisten with a small amount of water, add 1 tablet of Kjeldahl catalyst and 5 mL of concentrated sulfuric acid, then digest at 400 °C for 1 h. After digestion, cool to room temperature, dilute to 50 mL with distilled water, and prepare the test solution after filtration or clarification. Transfer 5 mL of the test solution into the distillation flask of the Kjeldahl apparatus. Add alkali to distill and release ammonia. Absorb the ammonia with boric acid solution, then titrate with standard sulfuric acid solution. Calculate crude protein content based on the nitrogen content using the nitrogen-to-crude protein conversion factor. This study employed a nitrogen-to-crude protein conversion factor of 6.25. This factor is widely used in plant seed protein determination and exhibits high compatibility with the actual amino acid composition of flaxseed protein, effectively reducing calculation errors in protein content estimation [20]. This method refers to the national standard, and no obvious modification was made. Crude protein content calculated by this method is an estimate based on total nitrogen content; non-protein nitrogen in flaxseeds and the assumed nitrogen-to-protein conversion factor may introduce a small amount of bias into the test results.

#### 4.2.2. Determination of Hydrolyzed Amino Acid Content

This study did not verify the crude protein content based on amino acid composition, which is one of the limitations of this research. Accurately weigh the sample, add 2 mL of 50% ethanol solution containing 0.1 mol/L HCl, subject to low-temperature ultrasonic treatment for 30 min, then centrifuge at 12,000 rpm for 5 min. Aspirate 1 mL of the supernatant, freeze it, freeze-dry it, redissolve it in 1 mL of amino acid sample diluent, filter it through a 0.22 μm membrane filter, and determine it using a Hitachi LA8080 amino acid analyzer [21,22] (Hitachi High-Tech Science Corporation, Tokyo, Japan). Instrument settings are as follows: injection volume 20 μL, indophenol flow rate 0.35 mL/min, reactor temperature 135 °C, mobile phase flow rate 0.45 mL/min, column temperature gradient from 57 °C to 74 °C, detection wavelengths 570 nm and 440 nm. The Hitachi LA8080 amino acid analyzer employs cation exchange chromatography for amino acid separation. Combined with post-column indophenol derivatization, it achieves baseline separation of 17 hydrolyzed amino acids with detection limits as low as 0.01 μmol/L, meeting the requirements for quantitative determination of trace amino acids [23]. This method refers to the published protocol, and no significant modification was made.

#### 4.2.3. Determination of Fatty Acid Components

Weigh a sample containing 100–200 mg of fat (accurate to 0.1 mg) into a flat-bottomed flask. Add pyrogallic acid, ethanol, water, and ammonia solution. Hydrolyze at 70–80 °C for 20 min. After hydrolysis, add ethanol. Extract the fat using a mixture of ether and petroleum ether. Concentrate the extract to dryness using a rotary evaporator. Add 2% sodium hydroxide in methanol to the fat extract and reflux at 80 ± 1 °C until oil droplets disappear. Add boron trifluoride in methanol and continue refluxing. After cooling, add n-heptane and saturated sodium chloride aqueous solution. Take the upper layer solution, dry it over anhydrous sodium sulfate, and determine it using a Thermo Trace 1300 gas chromatograph (Thermo Fisher Scientific, Waltham, Massachusetts, USA) [24]. Chromatography parameters are set as follows: Inlet temperature 260 °C, detector temperature 280 °C, carrier gas flow rate 1.0 mL/min. The column temperature program is as follows: 100 °C held for 13 min, ramped at 10 °C/min to 180 °C and held for 6 min, ramped at 1 °C/min to 200 °C and held for 20 min, then ramped at 4 °C/min to 230 °C and held for 10.5 min. This column temperature program effectively separates palmitic acid (C16:0) and stearic acid (C18:0) while preventing oxidative degradation of α-linolenic acid (C18:3) at high temperatures, ensuring the accuracy of fatty acid component quantification results [25]. This method refers to the national standard, and no obvious modification was made.

#### 4.2.4. Determination of Crude Fat Content

Weigh 3.0 g of the homogenized sample (accurate to 0.001 g) and transfer it into a filter paper cartridge. Dry the round-bottomed flask and the filter paper cartridge in an oven at 105 ± 2°C for 2 h, and weigh the mass of the round-bottomed flask (m_0_) after cooling. Weigh the sample into the filter paper cartridge (mass m_1_), and extract with anhydrous ether or petroleum ether until no oil trace remains (approximately 6–12 h). After solvent recovery, dry the round-bottomed flask in an oven at 105 °C for 2 h, weigh the mass (m_2_) after cooling, and calculate the crude fat content using the formula: $\mathrm{crude}\;\mathrm{fat}\;\mathrm{content}(\%)=\frac{m2-m0}{m1}\times 100$ [26]. The boiling range of anhydrous ether (30–60 °C) in the Soxhlet extraction method ensures sufficient extraction of fats while reducing the dissolution of non-lipid substances. The extraction time in this study was set to 8 h, which was determined based on the relative stability in this single environment resulting from the pre-experiment that “the mass change of the extraction flask was less than 0.001 g after 8 h” [27]. This method refers to the national standard, and no obvious modification was made.

#### 4.2.5. Determination of Trace Element Content

Accurately weigh the sample (to 0.0001 g) and place it in a PTFE digestion vessel. Add 3 mL of concentrated nitric acid and perform microwave digestion. The digestion program is as follows: heat for 10 min to 150 °C and hold for 2 min, then heat for 18 min to 240 °C and hold for 30 min. After digestion, transfer the digested solution to a 25 mL volumetric flask. Rinse the digestion vessel with 1% nitric acid, combine the rinse solution with the flask contents, and dilute to the mark. Mix thoroughly before analysis using a Thermo iCAP 7200 HS Duo Inductively Coupled Plasma Emission Spectrometer (Thermo Fisher Scientific, Waltham, MA, USA) [28]. Qualitative analysis was performed based on characteristic elemental spectral lines, while quantitative analysis was conducted using the external standard method. This method refers to the national standard, and no obvious modification was made.

### 4.3. Data Processing

All nutritional indicator data were statistically analyzed using SPSS Statistics 26.0 software. This study mainly adopted technical replication for sample detection, and no biological replication was set due to experimental conditions; the results of one-sample *t*-test are only for preliminary comparison of the tested materials. Means and standard deviations were calculated to reflect the central tendency and dispersion of the data. A one-way analysis of variance (ANOVA) combined with Duncan’s multiple range test was employed to analyze significant differences in the nutritional components of mature seeds among different flax germplasm lines, with a significance level set at *p* < 0.05. GraphPad Prism 10.1.2 software was employed to generate relevant charts and graphs, visually presenting the analysis results. Box plots clearly displayed the quartile distribution and outliers of the data, while the calculation of 95% confidence intervals in linear regression analysis more accurately reflected the strength of correlations among various nutritional indicators [29].

## 5. Conclusions

This study systematically evaluated the nutritional components of 30 flax germplasm resources from Northwest and North China, and clarified the genetic diversity of crude protein, amino acids, fatty acids, crude fat and mineral elements in flax seeds. Several high-quality core germplasms were screened, including high-protein germplasms, high α-linolenic acid germplasm, high-oil germplasms and Fe-Ca dual-enriched germplasm, which provide direct material support for flax quality breeding.

For flax breeding and practical processing, high-protein germplasms can be used to develop plant protein products such as protein beverages, high α-linolenic acid germplasm is suitable for functional oil extraction, and mineral-enriched germplasms can be applied in nutrition-enhanced food development. In addition, the relatively independent accumulation of protein and fat suggests that independent genetic improvement is needed for the two traits to achieve simultaneous enhancement.

This study has certain limitations due to the single-environment evaluation and minimal replication. Subsequent research should conduct multi-environment trials with more replications to verify the results, and combine molecular biology techniques to explore the genetic regulation mechanism of flax nutritional traits for more precise breeding.

## Figures and Tables

**Table 1:** Measured crude protein content of flax germplasm resources.

Germplasm Name	Sample Code	Crude Protein Content (g/100 g)	Germplasm Name	Sample Code	Crude Protein Content (g/100 g)
YY-01	A1	22.48	YY-16	A16	18.35
YY-02	A2	23.87	YY-17	A17	19.89
YY-03	A3	23.13	YY-18	A18	20.79
YY-04	A4	20.64	YY-19	A19	19.72
YY-05	A5	23.61	YY-20	A20	18.53
YY-06	A6	23.28	YY-21	A21	20.36
YY-07	A7	17.41	YY-22	A22	20.50
YY-08	A8	21.56	YY-23	A23	21.15
YY-09	A9	21.07	YY-24	A24	23.37
YY-10	A10	20.35	YY-25	A25	20.94
YY-11	A11	12.07	YY-26	A26	22.14
YY-12	A12	17.98	YY-27	A27	22.03
YY-13	A13	18.81	YY-28	A28	22.73
YY-14	A14	19.44	YY-29	A29	21.91
YY-15	A15	20.46	YY-30	A30	23.97
Quality Control Sample	-	-	Parallel Sample	-	-
GSB-12 (QC1)	QC1	17.47 (Total N: 2.79 g/100 g)	YY-20 (Parallel 1)	A20-1	18.12
GSB-12 (QC2)	QC2	17.23 (Total N: 2.76 g/100 g)	YY-20 (Parallel 2)	A20-2	18.53
GSB-12 (QC3)	QC3	17.54 (Total N: 2.81 g/100 g)	YY-30 (Parallel 1)	A30-1	23.15
Reference Value	-	2.79 ± 0.14 g/100 g (Total N)	YY-30 (Parallel 2)	A30-2	23.97

**Table 2:** Descriptive statistical results of crude protein content in flax germplasm resources.

Statistical Indicator	Value	Unit
Sample Size	30	pieces
Content Range	12.07~23.97	g/100 g (DW)
Mean	20.85	g/100 g (DW)
Standard Deviation (SD)	2.17	g/100 g (DW)
Coefficient of Variation (CV)	10.41	%
Median	20.80	g/100 g (DW)
Range	11.90	g/100 g (DW)

Note: *p* < 0.05 indicates significant differences among germplasms (one-way ANOVA).

**Table 3:** One-sample *t*-test results between high-protein germplasm resources and the overall average.

Germplasm Name	Sample Code	Crude Protein Content (g/100 g, DW)	Overall Mean (g/100 g, DW)	Difference (g/100 g)	t-Value	df (Degrees of Freedom)	*p*-Value	Standard Error of Mean (SE)	95% Confidence Interval (Lower~Upper)	Significance
YY-30	A30	23.97	20.85	3.12	8.96	29	<0.001	0.35	23.24~24.70	***
YY-02	A2	23.87	20.85	2.92	8.34	29	<0.001	0.35	23.16~24.58	***
YY-05	A5	23.61	20.85	2.76	7.82	29	<0.001	0.35	22.89~24.33	***

Note: *** indicates *p* < 0.001, highly significant (*p* < 0.001) difference.

**Table 4:** Statistics of essential and umami amino acid contents in flax germplasm resources (mg/100 g dry sample).

Amino Acid Category	Amino Acid	Mean (mg/100 g)	Standard Deviation (mg/100 g)	Coefficient of Variation (%)	Content Range (mg/100 g)	Germplasm with Highest Content	Germplasm with Lowest Content
Essential Amino Acids	Threonine (Thr)	12.3	2.8	22.76	7.0–21.5	YY-26 (A26)	YY-17 (A17)
	Valine (Val)	1.8	0.3	16.67	1.2–2.4	YY-11 (A11)	YY-04 (A4)
	Methionine (Met)	0.3	0.1	33.33	0.2–0.4	YY-19 (A19)	YY-04/08/15, etc.
	Isoleucine (Ile)	0.6	0.2	33.33	0.2–1.0	YY-12/23/28 (A12/A23/A28)	YY-06/08 (A6/A8)
	Leucine (Leu)	1.9	0.4	21.05	1.4–2.8	YY-27 (A27)	YY-15 (A15)
	Phenylalanine (Phe)	2.7	0.8	29.63	1.3–5.0	YY-23/28 (A23/A28)	YY-15/17 (A15/A17)
	Lysine (Lys)	0.7	0.2	28.57	0.5–1.4	YY-25 (A25)	YY-16/17/19, etc.
	Histidine (His)	2.8	0.7	25.00	1.8–3.9	YY-13/23/28 (A13/A23/A28)	YY-21 (A21)
Umami Amino Acids	Aspartic Acid (Asp)	17.8	2.2	12.36	14.2–23.2	YY-03 (A3)	YY-15 (A15)
	Glutamic Acid (Glu)	21.2	4.9	23.11	12.3–30.6	YY-05 (A5)	YY-17 (A17)

**Table 5:** Statistics of total amino acid content and ratios of core flax germplasm resources (mg/100 g dry sample).

Germplasm Code	EAA	NEAA	TAA	EAA/TAA (%)	EAA/NEAA (%)
YY-01 (A1)	0.0342	0.0513	0.0855	40.00	66.67
YY-03 (A3)	0.0371	0.0568	0.0939	39.51	65.32
YY-05 (A5)	0.0403	0.0632	0.1035	38.93	63.77
YY-11 (A11)	0.0385	0.0628	0.1013	38.01	61.31
YY-23 (A23)	0.0482	0.0735	0.1217	39.61	65.58
YY-25 (A25)	0.0432	0.0685	0.1117	38.67	63.07
YY-26 (A26)	0.0418	0.0587	0.1005	41.59	71.21
YY-27 (A27)	0.0456	0.0753	0.1209	37.72	60.56
YY-28 (A28)	0.0479	0.0741	0.1220	39.26	64.64
YY-30 (A30)	0.0398	0.0589	0.0987	40.32	67.57
Mean	0.0396	0.0612	0.1008	39.22	64.71
Maximum	0.0482	0.0753	0.1220	41.59	71.21
Minimum	0.0315	0.0489	0.0804	36.15	59.82

**Table 6:** Statistical analysis of the main fatty acid component contents in flax germplasm resources.

Fatty Acid Component	Mean (%)	Standard Deviation	Coefficient of Variation (%)	Content Range (%)	Germplasm with Highest Content	Germplasm with Lowest Content
α-linolenic acid	33.6	6.2	18.5	25.1~40.3	YY-15	YY-22
Linoleic acid	15.2	3.2	21.3	10.4~20.8	YY-09	YY-17
Palmitic acid	12.7	2.1	16.8	9.2~16.4	YY-03	YY-28
Stearic acid	7.4	1.0	14.1	5.7~9.3	YY-11	YY-26

**Table 7:** Fatty acid composition table of high α-linolenic acid germplasm resources.

Germplasm Code	α-Linolenic Acid Content (%)	Linoleic acid Content (%)	Oleic Acid Content (%)	Total Unsaturated Fatty Acids (%)	Total Saturated Fatty Acids (%)
YY-15	40.3	18.6	22.5	81.4	8.7
YY-06	38.7	16.2	24.1	79.0	9.2
YY-09	36.5	20.8	21.3	78.6	8.9
YY-03	34.9	15.3	23.7	73.9	10.1
YY-11	33.2	17.5	20.9	71.6	9.5

Note: Total unsaturated fatty acids = α-linolenic acid + linoleic acid + oleic acid + other trace unsaturated fatty acids; Total saturated fatty acids = palmitic acid + stearic acid + other trace saturated fatty acids. All data are calculated based on dry sample detection results.

**Table 8:** Statistical analysis of crude fat content in flax germplasm resources.

Statistical Dimension	Value (%)
Content Range	28.49~40.22
Mean	34.57
Standard Deviation	2.51
Coefficient of Variation	7.26
Median (50th Percentile)	34.56
25th Percentile (Q1)	33.39
75th Percentile (Q3)	36.21
Interquartile Range (IQR)	2.82
Mode Range	34.00~36.00
Proportion of High-Content Germplasm (≥36%)	33.33%
Proportion of Low-Content Germplasm (≤32%)	16.67%
Coefficient of Variation Comparison (with Total Protein)	1:1.38~1:2.07

**Table 9:** One-sample *t*-test results between high-fat germplasm resources and the overall average.

Germplasm	Crude Fat Content (%)	Difference from Mean (%)	Standard Error (s/√n)	t-Value	Degrees of Freedom (n-1)	*p*-Value	Significance Mark	Conclusion (α = 0.05)
YY-26	40.22	5.65	0.465	12.15	29	<0.001	***	Highly significantly (*p* < 0.001) higher than the mean
YY-22	39.16	4.59	0.465	9.87	29	<0.001	***	Highly significantly (*p* < 0.001) higher than the mean
YY-27	37.78	3.21	0.465	6.90	29	<0.001	***	Highly significantly (*p* < 0.001) higher than the mean
Overall Mean	34.57	-	-	-	-	-	-	Reference benchmark

Note: *** indicates a highly significant difference at *p* < 0.001.

**Table 10:** Descriptive statistical results of each element (mg/kg dry sample, Ca and Mg in g/kg).

Element	Mean	Standard Deviation	Coefficient of Variation (%)	Minimum Value	Maximum Value	Content Range
Fe	45.74	7.63	16.68	25.69	58.94	25.69–58.94
Zn	27.57	4.21	15.27	15.77	38.75	15.77–38.75
Ca	2.25	0.32	14.22	1.51	3.88	1.51–3.88
Mg	4.02	0.35	8.71	3.15	4.76	3.15–4.76

**Table 11:** Correlation analysis among elements.

	Fe	Zn	Ca	Mg
Fe	1			
Zn	0.26 (*p* = 0.16)	1		
Ca	−0.02 (*p* = 0.92)	−0.06 (*p* = 0.74)	1	
Mg	0.05 (*p* = 0.79)	−0.01 (*p* = 0.97)	0.42 (*p* = 0.02) **	1

Note: marked as ** when *p* < 0.01.

**Table 12:** Elemental content of three flax germplasm resources.

Sample Name	Content in the Sample			
	Zn (mg/kg)	Ca (g/kg)	Mg (g/kg)	Fe (mg/kg)
YY-10	36.87	2.66	4.48	55.69
YY-18	31.18	2.29	4.71	53.62
YY-30	25.83	2.88	3.89	58.94

**Table 13:** Information on 30 tested materials.

Code	Germplasm Name	Source
YY-01	12131-1-8-3	Xinjiang Yili Prefectural Academy of Agricultural Sciences (Yining, Xinjiang, P.R. China)
YY-02	1101-1	Guyuan Branch of Ningxia Academy of Agriculture and Forestry Sciences (Guyuan, Ningxia, P.R. China)
YY-03	Wuya 8	Wulanchabu Academy of Agricultural Sciences (Ulanqab, Inner Mongolia, P.R. China)
YY-04	63	Inner Mongolia Academy of Agriculture and Animal Husbandry Sciences (Hohhot, Inner Mongolia, P.R. China)
YY-05	Z34	Institute of Crop Science, Gansu Academy of Agricultural Sciences (Lanzhou, Gansu, P.R. China)
YY-06	Z129	Institute of Crop Science, Gansu Academy of Agricultural Sciences (Lanzhou, Gansu, P.R. China)
YY-07	C Long 8-140	Institute of Crop Science, Gansu Academy of Agricultural Sciences (Lanzhou, Gansu, P.R. China)
YY-08	94-14	Institute of Crop Science, Gansu Academy of Agricultural Sciences (Lanzhou, Gansu, P.R. China)
YY-09	Zhangya 2	Zhangjiakou Academy of Agricultural Sciences (Zhangjiakou, Hebei, P.R. China)
YY-10	H228	Institute of Crop Science, Gansu Academy of Agricultural Sciences (Lanzhou, Gansu, P.R. China)
YY-11	08051	Xinjiang Yili Prefectural Academy of Agricultural Sciences (Yining, Xinjiang, P.R. China)
YY-12	51	Inner Mongolia Academy of Agriculture and Animal Husbandry Sciences (Hohhot, Inner Mongolia, P.R. China)
YY-13	1139	Institute of Crop Science, Gansu Academy of Agricultural Sciences (Lanzhou, Gansu, P.R. China)
YY-14	NM-21-2	Inner Mongolia Academy of Agriculture and Animal Husbandry Sciences (Hohhot, Inner Mongolia, P.R. China)
YY-15	Longya 13	Institute of Crop Science, Gansu Academy of Agricultural Sciences (Lanzhou, Gansu, P.R. China)
YY-16	EA-87	Institute of Crop Science, Gansu Academy of Agricultural Sciences (Lanzhou, Gansu, P.R. China)
YY-17	0502-1-1	Guyuan Branch of Ningxia Academy of Agriculture and Forestry Sciences (Guyuan, Ningxia, P.R. China)
YY-18	EB-11	Institute of Crop Science, Gansu Academy of Agricultural Sciences (Lanzhou, Gansu, P.R. China)
YY-19	2012-55-11	Gansu Agricultural Vocational and Technical College (Lanzhou, Gansu, P.R. China)
YY-20	1301-59	Zhangjiakou Academy of Agricultural Sciences (Zhangjiakou, Hebei, P.R. China)
YY-21	Bayan 21	Zhangjiakou Academy of Agricultural Sciences (Zhangjiakou, Hebei, P.R. China)
YY-22	1450-35-1	Dingxi Academy of Agricultural Sciences (Dingxi, Gansu, P.R. China)
YY-23	0912-18	Institute of Alpine Crops, Shanxi Agricultural University (Datong, Shanxi, P.R. China)
YY-24	XB1	Institute of Crop Science, Gansu Academy of Agricultural Sciences (Lanzhou, Gansu, P.R. China)
YY-25	Z232	Institute of Crop Science, Gansu Academy of Agricultural Sciences (Lanzhou, Gansu, P.R. China)
YY-26	Z444	Institute of Crop Science, Gansu Academy of Agricultural Sciences (Lanzhou, Gansu, P.R. China)
YY-27	Z507	Institute of Crop Science, Gansu Academy of Agricultural Sciences (Lanzhou, Gansu, P.R. China)
YY-28	Z554	Institute of Crop Science, Gansu Academy of Agricultural Sciences (Lanzhou, Gansu, P.R. China)
YY-29	Longya 16	Institute of Crop Science, Gansu Academy of Agricultural Sciences (Lanzhou, Gansu, P.R. China)
YY-30	Longya 17	Institute of Crop Science, Gansu Academy of Agricultural Sciences (Lanzhou, Gansu, P.R. China)

## Data Availability

The original contributions presented in this study are included in the article. Further inquiries can be directed to the corresponding authors.
